# Predicting Postoperative Mortality in Neonates with Congenital Gastrointestinal Anomalies: Development of a Prognostic Scoring System

**DOI:** 10.3390/children12101313

**Published:** 2025-09-30

**Authors:** Filla Reviyani Suryaningrat, Eka Rizki Wulandari, Devatri Hudayari, Natasha Amalda Ediwan, Lulu Eva Rakhmilla, Fiva Aprilia Kadi, Aris Primadi, Tetty Yuniati

**Affiliations:** 1 Department of Child Health, Faculty of Medicine, Universitas Padjadjaran, Hasan Sadikin General Hospital, Bandung 40161, Indonesia; devatri_hudayari@hotmail.com (D.H.); nat.amalda@gmail.com (N.A.E.); fiva.kadi@unpad.ac.id (F.A.K.); aris.primadi@yahoo.co.id (A.P.); tettyyusuf61@gmail.com (T.Y.); 2 Faculty of Medicine, Universitas Padjadjaran, Bandung 40161, Indonesia; ekarwulandari@gmail.com; 3 Department of Public Health, Epidemiology and Biostatistic Division, Faculty of Medicine, Universitas Padjadjaran, Bandung 45363, Indonesia; lulu.eva.rakhmilla@unpad.ac.id

**Keywords:** congenital gastrointestinal anomalies, neonates, prognostic score, mortality, surgery

## Abstract

**Highlights:**

**What are the main findings?**

- The FILLA score, a four-parameter prognostic model (sepsis, mechanical ventilation, prematurity, and upper gastrointestinal anomalies), accurately predicts postoperative mortality in neonates with congenital gastrointestinal anomalies.
- Neonatal sepsis and the need for mechanical ventilation were the strongest independent predictors, reflecting critical illness severity prior to surgery.

**What is the implications of the main findings?**

- The FILLA score provides a simple, preoperative-only tool for early mortality risk stratification in neonates, especially valuable in resource-limited settings.
- This score can guide perioperative management, surgical counselling, and targeted family communication, and has potential for integration into digital decision-support systems.

**Abstract:**

Background: Congenital gastrointestinal anomalies (CGIAs) are the third most common congenital malformation globally, with a mortality rate reaching 39.8% in developing countries. Surgical intervention is often necessary for life-saving or corrective purposes. However, postoperative mortality in resource-limited settings can reach up to 50%. Identifying prognostic factors is essential to improve clinical management and inform family counseling regarding potential outcomes. Objectives: We aimed to develop a prognostic scoring system to predict postoperative mortality in neonates with CGIAs. Methods: This retrospective study analyzed medical records of neonates who underwent surgery for CGIAs between 2020 and 2024. Prognostic factors were identified using logistic regression analysis. Receiver operating characteristic (ROC) curves were used to determine optimal cutoff points for mortality prediction. Results: A total of 282 neonates were included; 121 (42.9%) died and 161 (57.1%) survived. Multivariate logistic regression identified sepsis, mechanical ventilation, prematurity, and upper gastrointestinal anomalies as independent predictors of mortality. A scoring system was developed, with a score > 3 yielding a sensitivity of 83.5% and specificity of 72.0%. The area under the ROC curve (AUC) was 0.840 (*p* < 0.001). Conclusions: We developed a simple and reliable scoring system to predict postoperative mortality in neonates with CGIAs, which may support clinical decision-making and family counseling.

## 1. Introduction

Congenital anomalies remain a major cause of neonatal morbidity and mortality worldwide, particularly in low- and middle-income countries (LMICs) [1,2,3]. Among these, CGIAs rank as the third most common anomaly, with a global prevalence of approximately 6.42% of live births and a mortality rate reaching 39.8% LMICs [1,3,4].

Despite advancements in neonatal care, CGIAs continue to pose a significant clinical challenge due to delayed diagnosis, limited access to advanced medical interventions, and high rates of postoperative complications such as sepsis, respiratory failure, and multiorgan dysfunction, particularly in LMICs [1,5]. In Indonesia, epidemiological data on CGIAs remain scarce. At Hasan Sadikin General Hospital, a national referral center in West Java, 166 neonates with CGIAs were treated between January 2022 and September 2024, indicating a considerable clinical burden.

Surgical intervention remains the mainstay of treatment for CGIAs, aiming to correct anatomical defects and improve survival outcomes [1,6]. However, not all neonates are eligible for surgery due to poor clinical conditions, including severe sepsis, pulmonary infections, or electrolyte imbalances [7,8]. Even among those who undergo surgery, mortality remains high in resource-limited settings, underscoring the urgent need for reliable tools to predict postoperative outcomes [1,9].

Prognostic scoring systems serve as valuable tools in neonatal care by enabling early risk stratification, guiding clinical decision-making, and facilitating communication with families. However, to date, there is no established prognostic model specifically designed to predict mortality in neonates with CGIAs undergoing surgery. This study aims to develop a simple, practical prognostic scoring system based on clinical parameters readily available in most LMICs hospital settings, to assist clinicians in improving perioperative management and outcomes.

## 2. Materials and Methods

### 2.1. Study Design and Sample

This was a retrospective cohort study conducted at Hasan Sadikin General Hospital, a tertiary referral hospital in West Java, Indonesia. Data were obtained from hospital medical records and the Neonatology Division’s registry, covering the period from January 2020 to December 2024.

### 2.2. Study Population

The study included neonates diagnosed with CGIAs who underwent their first surgical procedure during the study period. Inclusion criteria: (1) neonates diagnosed with CGIAs undergoing initial surgery and (2) availability of complete medical records. Exclusion criteria: (1) neonates who died within 24 h postoperatively and (2) patients referred or discharged against medical advice before completing treatment.

The minimum sample size was calculated using the formula for prognostic studies:

$$n=\frac{10\times k}{L}$$

where k is the number of predictors (13) and L is the estimated event proportion (mortality = 0.5). Thus, the required minimum was 260.

### 2.3. Study Variables

The prognostic variables in this study included both clinical and laboratory parameters available preoperatively. Gestational age was determined from the first day of the last menstrual period, antenatal ultrasound assessment, or postnatal estimation using the New Ballard Score. Birth weight was recorded immediately after delivery and further assess relative to gestational age using the Lubchenco growth chart to classify fetal growth as small, appropriate, or large for gestational age. Associated congenital anomalies were defined as non-gastrointestinal malformations identified clinically or radiologically. The need for mechanical ventilation was noted in neonates requiring either invasive ventilation or continuous positive airway pressure (CPAP). Neonatal sepsis was defined as culture-proven sepsis confirmed by a positive blood culture obtained prior to surgery [10]. Types of CGIAs were categorized by anatomical location relative to the ligament of Treitz. The type of surgical intervention referred to the first surgical procedure performed during hospitalization.

Laboratory parameters were also assessed. Electrolyte imbalance was defined as abnormal serum sodium, potassium, chloride, or calcium levels based on neonatal reference ranges [11]. Hypoalbuminemia was defined as serum albumin < 3.0 g/dL [12], while decreased hematocrit was defined as hematocrit < 40% [13]. Elevated C-reactive protein (CRP) was defined as a CRP concentration above the normal reference range for neonatal age and gestational status. Leukopenia was defined as a total white blood cell count <5000/mm^3^ [11,14].

### 2.4. Outcome Measures

The primary outcome was postoperative mortality, defined as all-cause death occurring during hospitalization following surgery.

### 2.5. Statistical Analysis

Data were analyzed using IBM SPSS Statistics version 29.0 for Mac (SPSS Inc., Chicago, IL, USA). Univariate and bivariate analyses were performed using the Chi-Square test. Variables with *p* < 0.25 in bivariate analysis were included in a multivariate logistic regression to determine independent predictors. Models were then calibrated using AUC analysis using the Hosmer Lemeshow test and discrimination value. Calibration was good if Hosmer Lemeshow test *p* ≥ 0.05, and discrimination was good if AUC > 0.8. Final models were then transformed into a scoring system using each variable’s coefficient (β) and standard error (SE). A cut-off point was determined via Youden’s Index.

## 3. Results

### 3.1. Participant Characteristics

From January 2020 to December 2024, there were 345 neonates diagnosed with CGIAs, admitted to the Department of Child Health Hasan Sadikin General Hospital. Among these patients, there were 63 excluded from the study, including 34 lacks data, 26 died before surgery, and 3 died within 24 h post operative. Therefore, there were 282 cases enrolled in the retrospective study.

A total of 282 neonates with CGIAs who underwent surgical intervention were included in this study. Of these, 121 (42.9%) died during hospitalization, and 161 (57.1%) survived. The subject characteristics from the study are presented in Table 1. The median birth weight was 2656 g (range: 1100–3900 g), and the median gestational age was 37 weeks (range: 28–41 weeks). The most common types of CGIAs included anorectal malformations (43.6%), esophageal atresia (13.1%), and intestinal atresia (12.8%).

In this study, the most common types of CGIAs were anorectal malformations (43.6%), esophageal atresia (13.1%), and duodenal atresia (12.8%). Table 2 summarizes the distribution of each type of CGIAs along with the corresponding survival and mortality outcomes.

### 3.2. Bivariate Analysis

Bivariate analysis identified several clinical variables significantly associated with mortality, including gestational age < 37 weeks (*p* = 0.004; OR 2.259), low birth weight < 2500 g (*p* < 0.001; OR 2.607), presence of associated congenital anomalies (*p* = 0.001; OR 2.396), need for mechanical ventilation (*p* < 0.001; OR 8.159), neonatal sepsis (*p* < 0.001; OR 11.584), upper CGIAs (*p* < 0.001; OR 3.287). Other laboratory parameters, such as electrolyte imbalance (*p* = 0.008; OR 2.213), elevated CRP (*p* = 0.164; OR 1.400), hypoalbuminemia (*p* = 0.218; OR 1.353), and decreased hematocrit (*p* = 0.192; OR 1.394), were also initially included for further analysis. The result of the bivariate analysis are summarized in Table 3.

### 3.3. Multivariate Logistic Regression

Multivariate logistic regression analysis identified four variables as independent predictors of postoperative mortality. Neonatal sepsis had the strongest association with death (AOR 6.84, 95% CI: 3.42–13.71, *p* < 0.001), followed by the need for mechanical ventilation (AOR 4.61, 95% CI: 2.54–8.37, *p* < 0.001). Prematurity and upper gastrointestinal anomalies were also associated with increased mortality risk, although the associations were borderline significant (AOR 1.96, 95% CI: 0.99–3.89, *p* = 0.055; AOR 1.83, 95% CI: 0.99–3.37, *p* = 0.051, respectively). These variables were included in the prognostic scoring system (Table 4).

### 3.4. Prognostic Scoring System Development

Each independent predictor was assigned a score based on the magnitude of its regression coefficient. Table 5 shows the final scoring system developed from the multivariate model. The total prognostic score ranged from 0 to 8. The logistic regression equation for predicting mortality was

Logit(p) = −1.758 + 2.566 × (Total Score)

To enhance clinical applicability and recall, the scoring system developed in this study was named the FILLA score, an acronym for Four Indicators Linked to Lethality in Abnormalities (Table 5). This name reflects the model’s foundation on four independent predictors of postoperative mortality: neonatal sepsis, mechanical ventilation, prematurity, and upper gastrointestinal anomalies.

### 3.5. ROC Analysis and Cut-Off Determination

**Figure 1:**
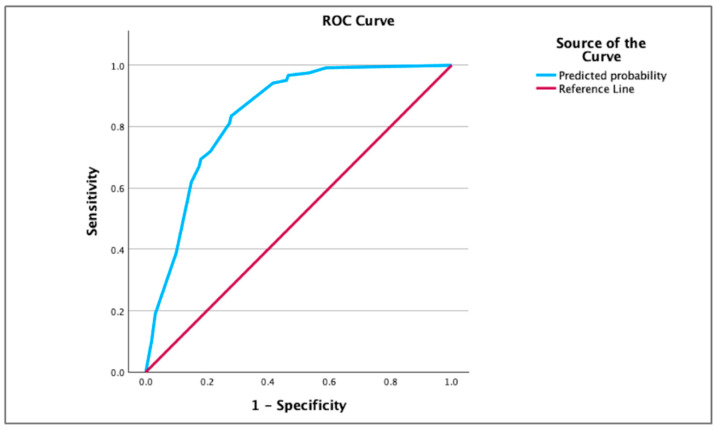
ROC curve. The scoring has a good discrimination (AUC = 0.840; CI 95% = 0.795–0.886; *p* < 0.001).

Receiver operating characteristic (Figure 1) analysis demonstrated strong discriminatory performance of the prognostic model, with an AUC of 0.840 (95% CI: 0.795–0.886; *p* < 0.001). Based on Youden’s Index, a cutoff score of greater than 3 was identified as the optimal threshold for predicting postoperative mortality. At this cutoff point, the model achieved a sensitivity of 83.5% and specificity of 72.0%. The positive predictive value (PPV) was 69.2%, while the negative predictive value (NPV) reached 85.3%. Furthermore, the positive likelihood ratio (LR+) was 2.98, and the negative likelihood ratio (LR-) was 0.23, indicating that the scoring system has good potential for clinical risk stratification and ruling out low-risk patients.

### 3.6. Risk Stratification

Based on the total prognostic score, patients were stratified into three risk categories to facilitate clinical decision-making. The predicted mortality percentages reflect modeled probabilities from logistic regression rather than raw proportions. This risk stratification framework may help clinicians allocate resources more effectively, initiate timely intervention, and conduct targeted family counseling based on objective prognosis estimates (Table 6).

## 4. Discussion

Congenital gastrointestinal anomalies are among the most common structural birth defects, ranking third in global prevalence after orthopedic and neurological anomalies [6]. Although the overall burden of neonatal mortality has declined in recent decades, CGIAs remain a major contributor to early postoperative deaths, particularly in resource-limited settings [15,16]. In Indonesia, the neonatal mortality rate in 2019 was 12 per 1000 live births, reflecting both improvements in perinatal care and the ongoing challenges in managing high-risk surgical conditions [17].

The postoperative mortality rate of 42.9% observed in this study is consistent with findings from other LMICs, where mortality associated with CGIAs frequently exceeds 40%, especially in settings without timely referral systems, adequate neonatal intensive care, or specialized surgical support [6,15]. Delays in diagnosis, sepsis, late-stage presentation, and limited perioperative infrastructure contribute substantially to these poor outcomes [6,18]. These persistent challenges underscore the urgent need for effective risk stratification tools to guide clinical decision-making and resource allocation in neonatal surgical care.

This study identified four independent predictors of postoperative mortality: neonatal sepsis, need for mechanical ventilation, prematurity, and upper gastrointestinal anomalies. Each of these factors reflects a critical clinical vulnerability that may inform targeted interventions. Sepsis emerged as the most significant predictor of mortality. With a six-fold increase in risk, sepsis exacerbates hemodynamic instability, induces multiorgan dysfunction, and impairs tissue repair following surgery [19,20]. Studies have shown that neonates with sepsis are more likely to develop postoperative complications such as wound dehiscence and prolonged ventilator dependence [10]. These findings are consistent with previous studies, which have consistently identified sepsis as one of the primary drivers of neonatal surgical mortality, particularly in LMICs where early diagnosis and infection control measures remain limited [5,6,21].

In our study, the diagnosis of sepsis was confirmed by the presence of a positive blood culture obtained prior to surgery, which, while specific, requires prolonged incubation time and may not be routinely available in many healthcare settings [22]. Early detection remains a major challenge [23]. Although laboratory-based sepsis markers such as elevated CRP, leukopenia, and hypoalbuminemia were explored, none showed statistically significant associations with mortality in our cohort [22,24]. This underscores the limitations of relying solely on these markers for early risk stratification, and the need for more robust, sensitive screening tools.

Our findings reinforce international recommendations advocating for prompt recognition and aggressive management of neonatal sepsis, particularly in resource-limited neonatal intensive care unit (NICU) settings. The timely initiation of broad-spectrum antibiotics, fluid resuscitations, and supportive care before surgical intervention may substantially improve postoperative outcomes [25,26]. Incorporating sepsis screening protocols into neonatal surgical pathways may serve as a critical step toward reducing mortality among this high-risk population.

Mechanical ventilation, although often essential for stabilization, was also independently associated with increased mortality [23]. The need for ventilatory support reflects the severity of respiratory compromise, which may stem from pulmonary immaturity, infection, or advanced disease at presentation. In this study, neonates who required mechanical ventilation had more than a fourfold increase in the odds of postoperative mortality. This finding is consistent with previous research, which also identified endotracheal intubation as a significant predictor of postoperative mortality in neonates. The requirement for mechanical ventilation frequently indicates the presence of severe respiratory distress, suggesting a reduced physiologic reserve and increased perioperative risk [5].

This study also aligns with findings from other study who reported that ventilator dependence was a strong predictor of poor outcomes in neonatal surgery, particularly in cases requiring prolonged support [21,23]. Mechanical ventilated neonates are often at increased risk for complication such as ventilator-associated pneumonia, oxygen toxicity, and barotrauma, which may further compromise recovery [27,28]. Additionally, ventilator dependence may reflect delayed presentation or more advanced disease severity at the time of surgery, both of which contribute to worse prognoses.

Prematurity, a known risk factor for poor outcomes in neonates, showed an adjusted odds ratio close to 2 in this study [21,29,30]. Preterm infants often have underdeveloped lungs, immature immune systems, and limited cardiorespiratory reserves, making them more vulnerable to perioperative complications [31]. They are at increased risk of sepsis, temperature instability, electrolyte imbalances, and delayed wound healing, all of which may impair recovery following surgery [21,29]. Moreover, their reduced physiological resilience often results in a lower tolerance for anesthesia and surgical stress, contributing to higher morbidity and mortality rates [32]. Although the association was only marginally significant in the multivariate analysis, the inclusion of prematurity in the scoring model is justified by its consistent association with adverse surgical outcomes in neonates, as shown in various global studies [6].

Upper gastrointestinal (GI) anomalies, including esophageal atresia and congenital diaphragmatic hernia (CDH), were associated with nearly a two-fold increase in mortality. These anomalies often require more complex surgical repair and are frequently accompanied by respiratory distress, feeding intolerance, or both [33]. Several studies have identified CDH as a major contributor to neonatal surgical mortality, largely due to severity of associated pulmonary hypoplasia and persistent pulmonary hypertension [34]. Surgical repair of diaphragmatic defects can be performed via either abdominal or thoracic access, with the abdominal route more commonly associated with gastrointestinal complication requiring reoperation [35].

Recent evidence further emphasizes that postoperative outcomes in CDH are strongly determined by the neonate’s baseline clinical condition rather than the surgical approach itself. A recent meta-analysis found no significant difference in morbidity between bedside and operating room repairs, noting instead that bedside procedures were typically performed in critically ill infants [36]. Similarly, a 2025 meta-analysis comparing thoracoscopic and open repair of CDH concluded that thoracoscopy was preferentially performed in neonates with lower morbidity, whereas open repair was more often applied in critically ill patients [37]. These findings support the concept that surgical decision-making for CDH is often driven by illness severity, which in turn explains the higher mortality observed in this subgroup.

Esophageal atresia has likewise been consistently recognized as a significant risk factor for mortality in neonates undergoing surgery [4]. These neonates often require meticulous preoperative stabilization and staged surgical correction and remain prone to complications such as anastomotic leaks, strictures, and aspiration pneumonia [23]. Beyond the perioperative period, a recent comprehensive review on long-term outcomes of CDH survivors emphasized persistent risks of chronic lung disease, gastrointestinal morbidity, and neurodevelopmental impairment, underscoring the broader burden of upper GI anomalies beyond the immediate postoperative phase [38]. Taken together, these findings support the inclusion of upper GI anomalies as a key prognostic factor in mortality risk models, as they consistently present with greater surgical complexity, higher preoperative instability, and more prolonged postoperative vulnerability compared to lower GI anomalies.

The scoring system developed in this study, the FILLA score, offers a pragmatic tool for early mortality prediction in neonatal surgical care. With an AUC of 0.840, it demonstrated comparable discriminatory performance to other neonatal scoring systems, such as the SNAP-II (AUC 0.894), but with fewer and exclusively preoperative variables [39]. This simplicity enhances its feasibility for bedside application in resource-limited settings. Potential integrations into neonatal care pathways includes use at NICU admission for risk stratification, preoperative surgical counseling, and early family discussion regarding prognosis. Furthermore, the FILLA score may be adapted into digital decision-support tools, such as mobile applications or electronic calculators, to facilitate rapid bedside assessment.

This study has several limitations. First, the cohort comprised a heterogeneous group of CGIAs, with varying severity and prognosis, which may introduce selection bias. Our aim, however, was not to compare outcomes between specific anomalies, but rather to identify broadly applicable preoperative predictors across the CGIAs spectrum. Second, neonates who died within 24 h postoperatively were excluded to reduce confounding from extreme perioperative instability; nonetheless, this exclusion may have led to an underestimation of the true perioperative mortality risk. Third, potentially relevant variables—including timing of presentation and surgery, nutritional status, antenatal diagnosis, socioeconomic background, and access to advanced NICU care—were not consistently available in our retrospective dataset. Fourth, surgical characteristics such as procedure type and duration, intraoperative blood loss, transfusions, and postoperative complications (e.g., anastomosis leak or surgical site infection) were not incorporated because of incomplete and inconsistent documentation. While this reduced granularity, our primary goal was to design a prognostic tool based solely on simple, readily available preoperative variables that can be applied in resource-limited settings. Future multicenter prospective studies with more comprehensive perioperative data collection are needed to validate the FILLA score and enhance its predictive accuracy and clinical utility.

## 5. Conclusions

We developed a simple and clinically applicable prognostic scoring system, the FILLA score, to predict postoperative mortality in neonates with congenital gastrointestinal anomalies. The score incorporates four independent predictors, neonatal sepsis, need for mechanical ventilation, prematurity, and upper gastrointestinal anomalies, and demonstrated good discriminatory performance. By relying exclusively on preoperative clinical parameters, the FILLA score provides a pragmatic tool for early risk stratification, guiding perioperative management, and supporting family counseling, particularly in resource-constrained settings. Prospective multicenter validation will be essential to confirm its generalizability, optimize risk thresholds, and potentially integrate the score into digital decision-support platforms for neonatal surgical care.

## Figures and Tables

**Table 1:** Subject characteristics of neonates with CGIAs who underwent surgery.

Variable	Died (n = 121)	Survived (n = 161)	*p*-Value	OR
Gestational age (weeks)				
Median (range)	37 (28–40)	38 (28–42)		
<32	4 (1.4%)	4 (1.4%)	0.374	1.891
32–37	62 (22.0%)	53 (18.8%)	0.001	2.212
>37	55 (19.5%)	104 (36.9%)		
Birth weight (g)				
Median (range)	2500 (1100–3900)	2800 (1100–3800)		
<1500	2 (1.0%)	2 (1.0%)	0.538	1.848
1500–2499	53 (19.1%)	37 (13.3%)	<0.001	2.648
≥2500	66 (34.4%)	122 (63.5%)		
Fetal growth				
SGA	13 (4.6%)	17 (6.0%)	0.932	1.017
LGA	5 (1.8%)	7 (2.5%)	0.965	0.950
AGA	103 (36.5%)	137 (48.6%)		
Sex				
Male	66 (23.4%)	104 (36.9%)	0.088	0.658
Female	55 (19.5%)	57 (20.2%)		
Referral status				
Yes	94 (33.3%)	136 (48.2%)	0.146	0.640
No	27 (9.6%)	25 (8.9%)		
Congenital heart disease				
Yes	29 (10.3%)	18 (6.4%)	0.004	2.504
No	92 (32.6%)	143 (50.7%)		

Abbreviations: SGA, small for gestational age; LGA, large for gestational age; AGA, appropriate for gestational age; n, number of patients; OR, odds ratio.

**Table 2:** Mortality in CGIAs.

Types of CGIAs	Died (%)	Survived (%)	Total (%)
Anorectal malformation	24 (8.5)	99 (35.1)	123 (43.6)
Esophageal atresia	32 (11.3)	5 (1.8)	37 (13.1)
Duodenal atresia	19 (6.7)	17 (6.0)	36 (12.8)
Gastroschisis	14 (5.0)	11 (3.9)	25 (8.9)
Annular pancreas	8 (2.8)	7 (2.5)	15 (5.3)
Jejunoileal atresia	9 (3.2)	1 (0.4)	10 (3.5)
Diaphragmatic hernia	3 (1.1)	7 (2.5)	10 (3.5)
Ileal atresia	4 (1.4)	4 (1.4)	8 (2.8)
Hirschsprung	1 (0.4)	4 (1.4)	5 (1.8)
Malrotation and volvulus	3 (1.1)	1 (0.4)	4 (1.4)
Omphalocele	1 (0.4)	2 (0.7)	3 (1.1)
Jejunal atresia	2 (0.7)	0 (0)	2 (0.7)
Hypertrophic pyloric stenosis	1 (0.4)	1 (0.4)	2 (0.7)
Congenital band	0 (0)	1 (0.4)	1 (0.4)
Duodenal web	0 (0)	1 (0.4)	1 (0.4)

**Table 3:** Bivariate analysis of each variable to mortality.

Variable	Died (n = 121)	Survived (n = 161)	*p*-Value	OR	CI 95%
Gestational age (weeks)					
<37	39 (13.8%)	28 (9.9%)	0.004	2.259	1.293–3.947
>37	82 (29.1%)	133 (47.2%)			
Birth weight (g)					
1500–2499	55 (19.5%)	39 (13.8%)	<0.001	2.607	1.569–4.332
≥2500	66 (23.4%)	122 (43.3%)			
Fetal growth					
SGA	13 (4.8%)	17 (6.3%)	0.965	1.017	0.473–2.188
AGA	103 (40.9%)	137 (54.4%)			
Fetal growth					
LGA	5 (2.0%)	7 (2.8%)	0.932	0.950	0.293–3.079
AGA	103 (40.9%)	137 (54.4%)			
Associated congenital anomalies					
Yes	44 (15.6%)	31 (11.0%)	0.001	2.396	1.398–4.109
No	77 (27.3%)	130 (46.1%)			
Mechanical ventilation					
Yes	83 (29.4%)	34 (12.1%)	<0.001	8.159	4.759–13.987
No	38 (13.5%)	127 (45.0%)			
Neonatal sepsis					
Yes	107 (37.9%)	64 (22.7%)	<0.001	11.584	6.106–21.974
No	14 (5.0%)	97 (34.4%)			
Types of CGIAs					
Upper	62 (22.0%)	39 (13.8%)	<0.001	3.287	1.980–5.458
Lower	59 (20.9%)	122 (43.3%)			
Types of surgery					
Emergency	59 (20.9%)	77 (27.3%)	0.877	1.038	0.648–1.664
Definitive	62 (22.0%)	84 (29.8%)			
Electrolyte imbalance					
Yes	102 (36.2%)	114 (40.4%)	0.008	2.213	1.219–4.017
No	19 (6.7%)	47 (16.7%)			
Leukopenia					
Yes	52 (18.4%)	70 (24.8%)	0.933	0.980	0.609–1.577
No	69 (24.5%)	91 (32.3%)			
Elevated CRP					
Yes	68 (24.1%)	77 (27.3%)	0.164	1.400	0.871–2.248
No	53 (18.8%)	84 (29.8%)			
Hypoalbuminemia					
Yes	75 (26.6%)	88 (31.2%)	0.218	1.353	0.836–2.187
No	46 (16.3%)	73 (25.9%)			
Decreased hematocrit					
Yes	45 (16.0%)	48 (17.0%)	0.192	1.394	0.846–2.298
No	76 (27.0%)	113 (40.1%)			

Abbreviations: CI, confidence interval; n, number of patients; OR, odds ratio.

**Table 4:** Multivariate logistic regression for postoperative mortality predictors.

Variable	AOR	95% CI	*p*-Value
Neonatal sepsis	6.84	3.42–13.71	<0.001 *
Mechanical ventilation	4.61	2.54–8.37	<0.001 *
Prematurity	1.96	0.99–3.89	0.055
Upper GI anomaly	1.83	0.99–3.37	0.051

AOR: Adjusted Odds Ratio; * *p* < 0.05.

**Table 5:** FILLA score in predicting postoperative mortality in neonates with CGIAs.

No	Predictor	Score
1	Neonatal sepsis	3
2	Mechanical ventilation	3
3	Prematurity	1
4	Upper GI anomaly	1
	Total score	8

**Table 6:** Risk stratification based on prognostic score and associated mortality.

Risk Category	Score	Predicted Mortality (%)
Low risk	0	14.7
Moderate risk	1	69.1
High risk	≥2	>96.7

## Data Availability

The original contributions presented in the study are included in the article; further inquiries can be directed to the corresponding authors.

## Abbreviations

The following abbreviations are used in this manuscript:

AGA	Appropriate for gestational age
AUC	Area under the curve
CDH	Congenital diaphragmatic hernia
CGIAs	Congenital gastrointestinal anomalies
CPAP	Continuous positive airway pressure
CRP	C-reactive proteins
FILLA	Four Indicators Linked to Lethality in Abnormalities
GI	Gastrointestinal
LGA	Large for gestational age
LMICs	Low- and middle-income countries
NICU	Neonatal Intensive Care Unit
ROC	Receiver operating characteristics
SGA	Small for gestational age
SNAP-II	Scores for Neonatal Acute Physiology-II
